# Disentangling the concept of “the complex older patient” in general practice: a qualitative study

**DOI:** 10.1186/s12875-016-0455-6

**Published:** 2016-06-03

**Authors:** S. A. Zwijsen, N. M. Nieuwenhuizen, O. R. Maarsingh, M. F. I. A. Depla, C. M. P. M. Hertogh

**Affiliations:** Department of General Practice and Elderly Care Medicine/EMGO Institute for Health and Care Research, VU Medical Center, Van der Boechorststraat, 71081 BT Amsterdam, The Netherlands; Verenso, the Dutch Association of Elderly Care Physicians and Social Geriatricians, Utrecht, Netherlands

**Keywords:** General practice, Qualitative research, Geriatrics, Ethics, Ageing, Comorbidity

## Abstract

**Background:**

The rising life expectancy in the developed world leads to an increase in the number of older patients and the complexity of their complaints in general practice. Although interventions and support for general practitioners are available, implementation lags. Knowledge on what determines a complex older patient, the problems of which general practitioners encounter and the situations they actually need support for, is necessary for better implementation.

**Methods:**

To provide support to general practitioners in their struggle with complex older patients, the aim of this research was to disentangle the concept of the complex older patient in general practice. A qualitative approach was used consisting of 15 semi-structured interviews with general practitioners. The general practitioner was asked to prepare a case of a complex older patient out of their own practice that could be discussed during the interview. Transcripts of the interview were analysed using inductive thematic analysis.

**Results:**

Analysis of the interviews resulted in twelve themes that could be categorised into five factors that contribute to the complexity of cases of older patients. The five factors are: not being in charge, different views on necessary care, encountering the boundaries of medicine, limits to providing social care, ill-equipped.

**Conclusion:**

The factors that were found imply that a better organisational structure for elderly care and consulting elderly care physicians could support general practitioners in providing care for older complex patients. Furthermore, understanding the current concept of patient autonomy seems unjustified in cases of complex older patients.

## Background

Due to improved living conditions, improved health care and more sophisticated medical treatment options, the number of older people in the developed world is rising quickly [1]. Although the life expectancy of the population is increasing, the later years are usually spent in imperfect health. Therefore, multiple medical, social and financial issues arise regarding the elderly [2, 3].

The concept of ageing-in-place, in which admittance to residential care is postponed as long as possible [4], is an answer to both the financial issue and the profound wish of today’s elderly to remain independent and autonomous [5]. One of the consequences of the ageing-in place concept in The Netherlands is that the elderly population is increasingly more scattered throughout the country instead of concentrated in several residential facilities. People with several comorbid complaints and functional disabilities (so-called complex patients) are now living at home instead of a residential facility. For general practitioners (GPs), this means an increase in the number of complex older patients they have.

Over the years, the issue of the increasing population of complex older patients in primarycare has become clear to both GPs and policymakers. Several attempts have been made to provide support for GPs. Interventions have been developed that focus on the management of complex care by structuring the approach to a disease or problem or providing support from other care disciplines [6–9]. Also, attempts have been made to improve collaboration between GPs and other health care disciplines like elderly care physicians and geriatricians.

The elderly care physician is a medical specialism unique in the Netherlands. Up until now, most elderly care physicians have worked in residential settings and nursing homes. Since more and more complex older patients are living at home, collaboration between elderly care physicians and GPs seems appropriate.

Elderly care physicians could thus help GPs in the management of complex older patients. To provide support for the GP in their struggle with complex older patients, knowledge is necessary on what determines the complexity of an older patient from the GP point of view. Until now, the term ‘complex’ has been used as a mere addition of several psychological, social and medical problems [10–13]. The lack of a clear definition of the term complex may have led to the current situation in which the interventions and collaboration between disciplines have not yet been optimally implemented [14].

Although there are several support options for the GP, like the involvement of a practice nurse or referral to medical specialists, these are not always realistic or preferred options. It is unlikely that every form of support fits every case of complex elderly care. Currently, no studies are available into what characteristics make a case of an older patient complex. As part of a larger study on the possible collaboration between elderly care physicians and GPs, in this paper we describe an explorative qualitative study that was conducted to disentangle the concept of ‘the complex older patient’ from the GPs point of view.

## Methods

### Research team and reflexivity

#### Personal characteristics researcher

The interviews were conducted by the female author NMN, who is an elderly care physician working as a physician and a researcher. She was trained by author CH, who has conducted and supervised qualitative research for over 20 years.

#### Relationship with participants

The researcher introduced herself as an elderly care physician, and explained before each interview that the goal of the research project was to study the possibilities for collaboration between general practitioners and elderly care physicians.

### Study design

#### Theoretical framework

Because no data on the problems GPs encounter with complex older patients was available, a qualitative approach with semi-structured interviews was chosen. Qualitative research is useful for exploring problems and questions related to rapidly emerging phenomena like the increasing population of complex elderly patients [15, 16]. Individual interviews are useful for obtaining more detail and personal experience about care issues with complex older patients [15]. Inductive thematic analysis was used to interpret the data, which means themes were derived from the data [17].

#### Participants selection and setting

To learn more about the problems GP encounter with complex older patients, purposeful sampling was used to select 15 Dutch general practitioners for interviews. The general practitioners were approached through the use of social media and connections that emerged from earlier research and practice experience. The participants were selected to represent the diversity in the patient population (rural background, ethnicity, age), and the GP population in gender and years of experience. Nine women and six men were interviewed with a median practice experience of 5–10 years, most of them held practice in a larger team of GPs (Table 1). The interviews took place at the general practice between March 2012 and May 2013. Each interview lasted approximately 50 min. After 11 cases, signs of saturation appeared and. After this, four more interviews were held to check whether data saturation was indeed achieved and no new information came at hand.

**Table 1:** Demographics of physicians *N* = 15

Women/men	9/6
Age	
<35	1
35–55	9
>55	5
Years of experience	
<5	1
5–10	8
11–20	2
>20	4
Urban/Rural	9/6
% Solo practice	20
% duo practice	33
% group practice/larger health center	46

#### Data collection

The GP was asked to prepare a case out of their own practice that could be discussed during the interview. The patient had to be a case considered exemplary for complex older patients. No criteria about the patient were used; practitioners were free to choose the exemplifying complex older patient.

The interviews were conducted on the basis of a topic list developed by the research team (two elderly care physicians, general practitioner and psychologist) (Table 2). The overlapping research question was ‘what makes a complex older patient complex for the GP?’. Every GP was interviewed once. All interviews were audiotaped and transcribed verbatim.

**Table 2:** Topic list

Introductory question (<1 week before interview)	Choose a patient who exemplifies the problems you experience in providing care for complex older patients in primary care.
Topics during the interview	Description of the patient case
	Motivation for choosing this case
	Description of functions of the patient e.g.; ADL, mobility, psychological, cognitive, social aspects
	Summarising the key problem
	Description of the action undertaken to treat the patient/solve the problem
	Description of the formal and informal care system around the patient
	Reflection on the own role of the GP in this case
	Assessing the means needed to properly treat this patient
	Assessing the (need for) (interdisciplinary) support
	Summary and verification

The methods of data collection and analysis were not modified during the study.

#### Data analysis

The transcripts were analysed using inductive thematic analysis [17]. Inductive analysis is a method for finding trends and patterns in qualitative data. Inductive thematic analysis is flexible because of the theoretical freedom; it is a useful research tool for interpreting data. In inductive analysis, no pre-supposed themes are used. Three researchers with ample experience in the field (NN, elderly care physician, MD, psychologist, OM, general practitioner) independently analysed the interviews. This way analysis benefitted from a multidisciplinary perspective. No software was used to pick out themes, but rather, the researcher formulated 2 key-questions that were found to be central to answering the main research question.

All interviews were analysed separately by the researchers using the 2 key-questions.

- What is the main problem in taking care of this patient for this GP?
- What would this GP need to adequately take care of this patient?

For answering the 2 key-questions the researchers coded the transcripts individually, and formulated answers per question using citations from the interviews. A meeting was held to reflect on the formulated answers. Secondly a researcher (NN) aggregated the answers within the case, and formulated 2–5 answers (problems as seen by the GP) that would fit the citations mentioned before. These results were then discussed in another consensus meeting resulting in the final formulation of the answer to the questions. It resulted in 3 to 5 different problems per case.

When al interviews were analysed a final consensus meeting between the three researchers took place, in which an overall cross-case analysis resulted in formulating the main problem areas in which general practitioners experience problems in caring for elderly complex patients in primary care. A fourth researcher (SZ, psychologist) then combined the emerged themes into categories which were discussed to reach consensus.

## Results

### Patient characteristics

Patients identified by the GP as an ‘exemplifying complex older patient’ were usually females with a Western cultural background (*N* = 12), over 85 years of age (*N* = 10), with multimorbidity (>2 chronic conditions; *N* = 15), with a risk of falling (*N* = 7). Often, the GP questioned the cognitive function of the patient (*N* = 5) or cognitive dysfunction was already diagnosed (*N* = 4). Seven patients had a (suspected) psychiatric diagnosis, and two had a mental impairment.

### Factors contributing to complexity

Analysis of the interviews resulted in twelve themes that could be categorised in five factors that contribute to the complexity of older patients (Table 3). These five factors (not being in charge, different views on necessary care, encountering the boundaries of medicine, limits to providing social care, ill equipped) are described below.

**Table 3:** Five factors that contribute to the complexity of cases with older patients

Factor	Theme
Not being “in charge”	No oversight on care delivered
	Lack of an efficient registration system
	Professional care is suboptimal
Different views on necessary care	Patient declines treatment
	Family members of the patient pressure the GP
Encountering the boundaries of medicine	GPs doubt the benefits of treatment
	Symptoms cannot be resolved.
Limits to providing social care	No informal care system
	Not enough time
	Not enough information on available social support options
Ill-equipped	Not enough knowledge on specific diseases
	No professional support for GP [referral options, support of specialised nurses]

**Box 1:** **Exemplifying quotes from the interviews**

1a. And when I make arrangements, well, I can write it down in the file for the home-carers, but then I encounter the problem of how to inform the physical therapist or the people of the day care centre, that kind of stuff
1b. Yes, there are different shifts and 15 different people are involved with one lady. So then they must have a team meeting and they all must understand how to approach such a person. And that’s just…well, I can see it’s not working
2a. Maybe she is becoming demented…she is suspicious… Well, may she? Yes, maybe an 88-year old woman is allowed to go through a slight character change…But well…It does go too far when she won’t accept visitors. But maybe I am seeing things too negatively
2b. Somebody who does not want anything has that right, so then you are trapped…While simultaneously you feel pressure from the family, pointing out that he is not doing well.
3. One and a half years ago, she went to see the cardiologist because of some valve problems, but no cause was found. Very frustrating […] You would think we have a cure. So I prescribe something, but she complains again.
4. My weekly attendance prevents escalation. […]. Yes, actually, I am over there too often […]. Well, really, there should be nursing professionals with more experience with Parkinson patients. That would reduce my presence to only once a month [instead of once a week].
5a. I thought you should stay mobile, especially when you have Parkinson’s disease you should practice that. But that was just a thought I had and I have no idea if it is actually true
5b. Can we make it possible for them to stay living in their own home (with M. Parkinson)? I don’t think the neurologist knows. I fear that a geriatrician also doesn’t know.

### Not being “in charge”

The complex older patient is often surrounded with a jumble of professional and informal carers. This can be confusing for the patient and GP. GPs highlighted the lack of an efficient registration system in which all involved health professionals can communicate about the patient (Box 1: 1a). Although the GP tries to coordinate care surrounding the complex older patient, the amount of independently operating carers leads to a feeling of not being in charge of the care delivered. At the same time, GPs feel that they are responsible for their patients. The feeling of being responsible whilst not “in charge” creates frustration and a feeling of inadequacy. This feeling is intensified when the quality of professional care is suboptimal in terms of education, staff turnover and available time (Box 1: 1b).

### Different views on necessary care

Although GPs might have a clear idea of which intervention would be beneficial for their patients’ health, the view of the GP is not always the leading and conclusive one. For instance, some patients have psychosocially based problems (i.e. loneliness, isolation), while potential solutions for the problems (i.e. attending day care programmes) are rejected. Some older patients resist interventions, claiming they are too old or that taking pills or using assistive equipment is a hassle. When patients decline treatment, GPs often suspect the patient of having dementia, which complicates the matter even further (Box 2: 2a). The GP and patient can agree on no treatment, whilst family members of the patient pressure the GP to ‘do something’ (Box 2: 2b). When different views on what is best for the patient arise, the GP is torn between being responsible for providing adequate treatment, giving in to concerns of family members and wanting to respect the autonomy of the patient (Box 2: 2a).

### Encountering the boundaries of medicine

Generally, patients visiting a general practice seek help for their health care needs and medical problems. Although sometimes ‘watchful waiting’ is the appropriate treatment, usually a treatment option is available when symptoms increase or health deteriorates. When taking charge of the health of a complex older patient, however, the GP is confronted with many cases in which they feel they cannot provide adequate treatment. For some treatments or diagnostic procedures, GPs doubt whether the benefits outweigh the burden for the already frail patient. They believe many symptoms in old age simply cannot be alleviated, which seemed to frustrate some of the GPs (Box 3). GPs stated that they often feel empty-handed because the patient is in obvious need of help while the GP cannot provide medical treatment for their symptoms.

### Limits to providing social care

When older patients don’t have an informal support system, problems arise when patients are unable to travel or walk longer distances and GPs worry that important health problems are not reported. GPs question themselves to what extent they should be responsible for providing social care (Box 4). Many GPs have insufficient time for their complex older patients because their own full schedules don’t permit social visits to patients or a patient demands excessive time. GPs state that they suspect there are other professionals who can provide social support, but they lack accurate knowledge on the available psychosocial support in the area.

### Ill-equipped

GPs stated they often feel they are not optimally prepared to care for complex older patients. They have insufficient knowledge on the treatment of specific diseases of old age, like Parkinson’s disease or challenging behaviour in dementia (Box 5: 5a). GPs state they find it difficult to get full insight into the whole situation and the possible interventions or support options for the patient and they feel like they are alone in trying to come to grips with complex older patients (Box 5: 5b).

## Discussion

The aim of this study was to disentangle the concept of the complex older patient in general practice. *Not being in charge, different views on necessary care*, *encountering the boundaries of medicine*, *limits to providing social care* and *feeling ill equipped* all contribute to the complexity of such cases. The selection of cases also implies certain patient characteristics that add to complexity. Most cases were of patients in the age over 85 with multi-morbidity. Apparently, the combination of old age and multi-morbidity is a relevant factor for a case becoming complex.

The results show the reasoning of GPs as to why and when care for older patients is experienced as complex. The themes ‘not being in charge’, ‘limits to providing social care’ and ‘feeling ill-equipped’ point to a lack of oversight and structure in the health care system for patients with care needs and an unstable or failing support system. This lack of oversight is not only experienced by GPs, but also by patient themselves as research of Latafortune and colleagues point out [18]. For GPs, this is frustrating for they feel pressured because they are responsible for their patients, but they don’t have control over the other care professionals involved. They lack insight into the competencies of these care professionals or don’t know who to consult for questions related to the more advanced stages of chronic diseases in old age. In these situations, taking the lead and coordinating care becomes too complex for the GP.

It is not surprising that most complex older patients are over 85 years of age, for these elderly are particularly at risk for having a no support system due to frailty or deaths of relatives and friends of their own age. This problem increases now that frail older persons are increasingly living in the community while relatives are living in other parts of the country. As other research pointed out earlier [18], it seems prudent to develop a communication and information system for care issues involving such complex older patients as part of a solution to this problem. Smart-home technical devices that increase safety and help monitoring frail people might alleviate some of the pressure on GPs. Also these systems can help improve care by supporting people in managing their illness themselves [19], which is one of the pillars of the Chronic Care Model. However, what is most needed is multidisciplinary expertise regarding advanced chronic disease and care dependency. Until now, this expertise is limited to the nursing home setting and in The Netherlands the elderly care physician is still rarely consulted by GPs.

In the themes, ‘different views on necessary care’ and ‘encountering the boundaries of medicine’, GPs express frustration about being unable to treat a patient or to intervene in a situation. The amount of cases with multi-morbidity that was discussed illustrates the difficulty GPs have in determining what is the ‘best’ treatment for a patient. Research tends to be done on single diseases, which makes it unclear how to treat a patient with multiple diseases at the same time. This often leads to conflicts in decision making when a GP tries to follow best practice guidelines. Next to this, it seems that frustration arises where GPs believe they reached the limits of medicine, wherein they see no medical treatment for the patients’ problem or a patient declines the medical treatment proposed. In the former case, what is experienced as a boundary of medicine might also be viewed as a need for a change in perspective, away from a disease- and problem-oriented approach in favour of a focus on the consequences of disease, thus requiring other medical expertise. The geriatrician Mary Tinetti stated [20]: “concentrating on diagnosing the disease for which often little can be done can lead to ignoring or underplaying symptoms or disabilities for which often much can be done”. Regarding declining patients, GPs struggle with their own beliefs about appropriate treatment and how to respect the autonomy of the elderly. They believed that – in the end – the ‘right to say no’ must be respected provided that the patient does not suffer from decision making disability. Here, an approach to autonomy from the perspective of geriatric ethics might offer alternative strategies. For example, Agich [21] argues that autonomy and dependency are inextricably bound to each other, especially in old age. Following this view, respecting autonomy can also mean providing a person with enough support to maintain a feeling of integrity in that person’s most valued areas of life [21]. This may mean that, paradoxically, a physician sometimes needs to persuade a seemingly unwilling patient to undergo a treatment or intervention that ultimately leads to finding a new sense of autonomy.

Even with this approach to autonomy, GP’s have to weigh which value is most important in every individual case; the right to decline treatment or the right to receive treatment that could possibly enhance the patient’s autonomy. This is a complicated moral decision, especially in situations where family is involved in the decision making. To optimise such a decision, GP’s might benefit from consultations with an elderly care physician or an old age psychologist.

### Limitations

There are some limitations to this research. First, the sample of GPs was purposely selected to represent the variety in the population of patients, but there seems to be an underrepresentation of male, immigrant or foreign patients. It is, therefore, questionable whether all possible variations of the topic were explored. Also, GPs chose the cases they wanted to discuss. This may have resulted in cases that were rather extreme and do not represent the full spectrum of complex older patients.

Second, this study took place in the Netherlands and the patients and GPs represent the Dutch situation. Although there are other European care systems like the Dutch system, generalizebility to other countries questionable.

Third, the interviewing researcher is an elderly care physician herself. This might have introduced bias as the interviewer herself had ample experience with complex older patients and may therefore elicited certain specific responses from the GPs. However, the data was analysed by both psychologists, elderly care physicians and GPs, which should have eliminated this bias.

## Conclusion

Despite the limitations, this study was able to disentangle the umbrella term of the ‘complex older patient’. The results imply that structuring the organisation of care and consulting elderly care physicians or geriatricians could support GPs in providing care for the continuously growing group of older complex patients. The results also imply that developing one care model for all complex older patients is an illusion. Improvements in care for this population should focus on tailored care and formulating an individual care goal rather than creating a ‘one size fits all’ solution [22].

Whether all GPs recognise these problems is an important question for quantitative follow up research. Future research could also focus on developing and testing a toolkit that tackles the issues that were derived from this research.

The study was well prepared in consulting several experts before finalising the topic list and the several steps of the analysis process. Finally, the involvement of a GP, an elderly care physician, and psychologists ensure careful interpretation of the data and reliability of the results.

## Abbreviation

Gp, general practitioner.
